# New methods of nuclear medicine in thyroid

**DOI:** 10.1186/1756-6614-8-S1-A14

**Published:** 2015-06-22

**Authors:** Grzegorz Kaminski

**Affiliations:** 1 Military Institute of Medicine, Warsaw, Poland

## 

Since radioiodine was first introduced in the therapy of hyperthyroidism in the 1940s, in the 21^st^ century, thyroid has become the arena of development of nuclear-molecular biology imaging. SPECT and PET technics allow the visualization of small particles like peptides and their receptors. In PET with ^18^F-FDG we can assess metabolic activity of thyroid tumours. If there is higher metabolic activity, the tumour is more aggressive and the prognosis poorer. These novel methods let us observe the primary lesion and metastatic processes in iodine avid differentiated thyroid cancer (DTC) and medullary thyroid cancer (MTC). Potentially, each particle triggered with a radioisotope which is involved in a cell structure and/or its metabolism can be useful in molecular imaging. The first group of molecules used in radioisotope molecular imaging is peptide receptors agonists and antagonists. Somatostatin receptors are overexpressed in DTC and MTC. Therefore, somatostatin analogues triggered with radioisotopes are used in either imaging (^99m^Technetium, ^111^Indium, ^68^Gallium) or treatment (^90^Yttrium, ^177^Lutetium) of these malignancies. Implementation of appropriate chelator allowed the creation of radiopharmaceuticals conjugated with either SPECT or PET isotopes. It seems that the best method for visualization of MTC is PET with ^18^F-DOPA up till now. Recently, new radiolabelled tracers for MTC visualizations are under investigation: cholecystokinin – 2 (CKK-2) gastrin receptor ligand radiolabelled with ^111^Indium and glucagon – like peptide -1 (GLP – 1) labelled with ^99m^Technetium.
